# Supplementary material for: Pectoralis minor length index at 1 month postoperative can predict homolateral neuropathic pain 4 months after mastectomy with lymph node resection
Source: PLoS One. 2025 Jun 12;20(6):e0326119. doi: 10.1371/journal.pone.0326119 (PMC12161585; doi:10.1371/journal.pone.0326119)
Supplement: S1 Appendix — (PDF) [file pone.0326119.s001.pdf]

<Exercise description>

As the chest muscles can be shortened after breast cancer surgery, you may feel pain or discomfort during arm movements. Thus, you may feel difficulty during arm elevation, or during the weight lifting. To minimize this complaints, chest muscles stretching and scapular exercises are recommended.

1. **Chest muscles stretching (Target muscles: pectoralis major and minor)**

As the chest muscles mobility improves, the arm movement and range of motion are increased.

1) Lie on your sound side (non-operated side).
2) Gently pull back your shoulder (to retract your shoulder blade).
3) Hold the position for 10 seconds and bring back to the start position.
4) Repeat 10 times.
5) Perform this exercise twice daily, at least three times a week.

Video link:

2. **Scapular exercise**

You should move the scapular to backward and inward.

As the strength stabilizing the scapular to the trunk improved, the arm movement is improved.

1) Sit on the chair with your feet on the ground.
2) Slightly bend your both elbow then squeeze the both scapulars (blade bones) at back.
3) During the squeezing, you should feel the closeness of the two scapulars not elbow.
4) Perform this exercise twice daily, at least three times a week.

Video link:
