# Supplementary material for: Pectoralis minor length index at 1 month postoperative can predict homolateral neuropathic pain 4 months after mastectomy with lymph node resection
Source: PLoS One. 2025 Jun 12;20(6):e0326119. doi: 10.1371/journal.pone.0326119 (PMC12161585; doi:10.1371/journal.pone.0326119)
Supplement: Attachment — Submitted filename: 0. Response to Reviewers.docx [file pone.0326119.s006.docx]

**Journal Requirements:**

**Comment 1**

When submitting your revision, we need you to address these additional requirements. Please ensure that your manuscript meets PLOS ONE's style requirements, including those for file naming.

**Response:**

Thank you for your comment. The manuscript has been formatted appropriately including supporting information captions. File names have been updated according to the guidelines (e.g., “fig 1.tif”).

**Comment 2**

We note that there is identifying data in the Supporting Information file <study data>. Due to the inclusion of these potentially identifying data, we have removed this file from your file inventory.

The following are examples of data that should not be shared:

-Name, initials, physical address / Ages more specific than whole numbers / Internet protocol (IP) address / Specific dates (birth dates, death dates, examination dates, etc.)

-ID numbers that seem specific (long numbers, include initials, titled “Hospital ID”) rather than random (small numbers in numerical order)

Please remove or anonymize all personal information (ID), ensure that the data shared are in accordance with participant consent, and re-upload a fully anonymized data set. Please note that spreadsheet columns with personal information must be removed and not hidden as all hidden columns will appear in the published file.

**Response:**

Thank you for your comment. The ID is randomly generated by a computer software; however, the study ID has been reassigned to smaller numbers as per the comments. Therefore, the current version of the **study** data is fully anonymized, and there are no hidden spreadsheet columns.

**Comment 3**

Please include captions for your Supporting Information files at the end of your manuscript, and update any in-text citations to match accordingly.

**Response:**

Thank you for your comment. Captions for the supporting information have been attached at the end of the manuscript file. As per the guidelines, the previously attached **study data** file has been deleted, and the repository URL has been uploaded separately.

(Page 32, line 512 – 513) “The data supporting the findings of this study are openly available in Zenodo at http://doi.org/10.5281/zenodo.14863395.”

**Comment 4**

Please ensure that the English version of the IRB is signed by the chairperson and clearly stating the start and end dates of the approval effective period in a similar way to the Korean version of the IRB (from 2021-07-29 to 2022-07-28). In addition, the date next to the missing signature should be the same or after the approval date (2021-09-07).

**Response:**

Thank you for your valuable comments. The chairperson's seal has been added to the English version of the IRB. The study approval date matches the date next to the signature, and the effective period is identical in both the Korean and English versions.

**Comment 5**

Please ensure that the study registration status (+/- the registration platform and the registration number) is clearly stated in the methodology section.

**Response:**

Thank you for your valuable comments. We have now added the registration platform and registration number in the Material and Methods section as requested. (Page 6, line 92 – 94) “The study was registered with the Clinical Research Information Service prior to participant enrollment (Trial registration number: KCT 0006501).”

**Comment 6**

Please ensure that a well-explained participant flow-chart is included as a diagram or figure in the methodology section.

**Response:**

Thank you for your valuable comments. We have inserted the study flowchart in the Materials and Methods section and labeled it as **Fig 1**. (Page 7, line 126 – 128)

**Comment 7**

Please ensure that all references in the list of references follow the Vancouver styling and are properly formatted.

**Response:**

Thank you for your comments. The manuscript follows the preferred style and the references have been properly formatted accordingly. (Page 34 – 38)

**Reviewer #1:**

Thank you for your valuable comment. We believe that the manuscript has been improved as per your recommendations. In accordance with all reviewers’ comments, statistical analysis was conducted again with robust manners.

**Comment 1**

Although 135 patients were screened, more than half were excluded. The reasons for this should be stated.

**Response:**

Thank you for your comments. The relevant details (reasons for exclusion) have been described in the Results section (Page 13, line 244 – 245):

“Of the 75 excluded patients, 19 declined participation while the remaining 56 were excluded for medical reasons (Fig 1).”

These exclusions are also illustrated in the study flowchart (**Fig 1**). (Page 7, line 126 – 128)

**Comment 2**

It appears that rehabilitation started one month after surgery, but what kind of pain relief medication was prescribed at the one-month mark?

**Response:**

Thank you for your comments. The relevant details have been described in the Methods section (Page 13, line 246 – 253).

“Among the 60 evaluated patients, 18 received pain medication during follow-up. Seventeen patients were prescribed nonsteroidal anti-inflammatory drugs, including 15 who received aceclofenac as part of their chemotherapy regimen and two who were prescribed celecoxib at the Department of Hematology and Oncology Clinics. Of the aceclofenac recipients, two patients were also prescribed gabapentin and tramadol, respectively at the Department of Plastic Surgery Clinic 1 month postoperatively. Furthermore, one patient received only gabapentin at the Department of Plastic Surgery Clinic 1 month postoperatively.”

**Comment 3**

This study investigates the prediction of NP four months after surgery based on data from one month post-surgery. What kind of rehabilitation program was implemented for the subjects during those three months? Also, was any pain relief treatment administered during those three months? These are important pieces of information in this study focused on NP.

**Response:**

Thank you for your comments. The relevant details regarding the rehabilitation program have been described in the Materials and Methods section. (Page 7, line 117 – 126).

“Participants were scheduled for physical therapy sessions. A.K. provided education and printed materials on pectoralis minor muscle stretching and scapular retraction exercises to all participants during the first educational session (S1 Appendix). Among the participants, 36 patients attended physical therapy for a median of three extra sessions (interquartile range [IQR]: 2–4 sessions), per the prescribed treatment plan. During these additional sessions, A.K. guided patients through shoulder elevation ROM exercises and administered deep frictional massage around the pectoralis minor muscles. Furthermore, 13 participants were prescribed self-care education sessions focused on edematous management, with a median of three sessions (IQR: 1 – 4 sessions), all conducted by a specialized physical therapist. Patients did not receive physical therapy such as thermoelectrical modality, to reduce pain.”

We believe that the ambiguity between neuropathic pain and postoperative pain at 1 month postoperatively may have led physicians to hesitate in aggressively treating pain at 1 month follow-up. Except pain medication, hot modality or electrical stimulation is typically not applied due to lymphedema and tumor infiltration risk. Although not explicitly mentioned, patients experiencing pain 4 months postoperatively received analgesics and appropriate treatment.

For details regarding pain relief medication, please refer to the Comment 2.

Pain medication during follow-up was included as a independent variable in the analysis. (Page 11, line 213 – 217)

**“**Given the relatively small sample size, a two-step logistic regression analysis was conducted to identify predictors. First, univariate logistic regression analyzed independent variables including age, body mass index, postmenopausal status, type of lymph node surgery, type of reconstruction, type of adjuvant therapy, pain medication during follow-up, and results from physical and questionnaire-based examinations.”

**Comment 4**

On page 23, it states, "Given the role of acute pain in NP development, the observed inconsistencies between our univariate and multivariate models are plausible." The reviewer does not understand this meaning. Could the differences between univariate and multivariate analysis results simply be due to sample size issues?

**Response:**

Thank you for your insightful comments. The previous sentences have been deleted. The original description aimed to emphasize that acute pain is an important predictor, although it was not selected in the multivariate regression model. In line with your valuable feedback, ambiguous statements have been removed, and the discussion section has been revised to more clearly highlight the relationship between pain and changes in pectoralis minor length. Aside from the small sample size, the results appear to be distinct from those influenced by other covariates, which have now been acknowledged in the study limitations. (Page 31, Line 480 – 483)

“Owing to the small sample size, the number of variables included in the model was also limited. To address this, we implemented a two-step logistic regression to identify effective predictors and covariates, during which ordinal variables, such as patient age, were excluded as part of the model selection strategy.”

**Reviewer #2:**

Thank you for your valuable comment. We believe that the manuscript has been improved as per your recommendations. In accordance with all reviewers’ comments, statistical analysis was conducted again with robust manners.

**Comment 1**

A flowchart should be added to the manuscript to enhance clarity and provide a visual summary of the study methodology.

**Response:**

Thank you for your comment. We have inserted the study flowchart in the Methods section and labeled it (**Fig 1**). (Page 7, line 126 – 128)

**Comment 2**

The sample size (N=57) was relatively small, which may limit generalizability and statistical power.

**Response:**

Thank you for your comment. We revised the sentences as per your comments. (Page 12, line 235 – 239)

“Sample size calculations and post-hoc power analysis were conducted using G*power software (latest version 3.1.9.7; Heinrich-Heine-Universität Düsseldorf, Düsseldorf, Germany). The power analysis determined that at least 42 participants would be required (OR: 3 [11]; α = 0.05; power = 0.8; and probability of Y: 0.5 [6]). Therefore, the final sample size was sufficient to run this analysis.”

**Comment 3**

Multivariate analysis only considers variables with p < 0.10 in univariate analysis, which might overlook potential confounding factors.

**Response:**

Thank you for your valuable comment. Following your recommendations, we have reestablished the threshold as 0.15. In accordance with the reviewer’s comments on statistical analysis, we have reconducted the analysis to ensure robustness. (Page 11, line 217 – 218) “Variables with a p-value < 0.15 in the univariate analysis were included in forward multivariate logistic regression.”

Therefore, the relevant analysis results have been added to the Results section, and table3 has been revised.

(Page 17, 283 – 299)

“Other predictors included the PMI (OR: 0.185; 95% CI: 0.046 – 0.745, p = 0.018), the strongest pain intensity during the past 4 weeks (OR: 1.311; 95% CI: 1.023 – 1.681, p = 0.032), the average pain intensity over the past 4 weeks (OR: 1.390; 95% CI: 1.010 – 1.915, p = 0.044), current pain intensity (OR: 1.369; 95% CI: 0.982 – 1.908, p = 0.064), quality of life (OR: 0.977; 95% CI: 0.950 – 1.005, p = 0.110), external rotation (OR: 0.968; 95% CI: 0.931 – 1.007, p = 0.111), arm symptoms (OR: 1.028; 95% CI: 0.993 – 1.064, p = 0.115), breast symptoms (OR: 1.025; 95% CI: 0.993 – 1.057, p = 0.129), pain medication during follow-up (OR: 1.975; 95% CI: 0.818 – 4.767, p = 0.130), physical function (OR: 0.971; 95% CI: 0.935 – 1.009, p = 0.131), Quick-DASH score (OR: 1.026; 95% CI: 0.991 – 1.061, p = 0.145), body mass index (OR: 0.844; 95% CI: 0.670 – 1.062, p = 0.147), abduction (OR: 0.987; 95% CI: 0.969 – 1.005, p = 0.149), and emotional function (OR: 0.976; 95% CI: 0.944 – 1.009, p = 0.149). When HLNP was redefined as a PDQ score ≥ 19, PMI approached statistical significance (OR: 0.177, 95% CI: 0.029 – 1.095, p = 0.062), followed by current pain intensity (OR: 1.408; 95% CI: 0.993 – 2.125, p = 0.103), pain subscale from the breast cancer-specific questionnaire (OR: 1.029; 95% CI: 0.994 – 1.065, p = 0.106), and radiation therapy history (OR: 3.083; 95% CI: 0.667 – 14.257, p = 0.064). PMI and current pain intensity exceeded the statistical threshold in both univariate regression analyses.“

**Comment 4**

No power analysis was reported to determine if the sample size is sufficient for detecting meaningful differences.

**Response:**

Thank you for your comment. Post-hoc power analysis was conducted using G*Power, and the relevant results are described in the Results section. Given the low power for systemic therapy side effects, the interpretation has been revised accordingly, and this limitation has been included as an additional study limitation.

(Page 22, line 318 – 322) “The post-hoc power analysis indicated power as 0.05 (OR: 1.056, α = 0.05, n = 57, probability of Y = 0.38) for ST. For PMI, the power reached 0.99 (OR: 4.9, calculated as 1/0.204). Similar to the univariate regression results, both factors require careful interpretation due to their modest ORs and wide confidence intervals.”

(Page 22, line 328 – 329) “Post-hoc analysis reported a power of 0.99 with an OR of 8.62 (1/0.116), α = 0.05, sample size = 57, and probability of Y = 0.14.”

(Page 32, line 478 – 480) “The small sample size contributed to the wide confidence interval for the OR of PMI and the significant but very low OR of ST, both of which reduced the statistical power of our analyses.”

**Reviewer #3:**

Thank you for your valuable comment. With your thorough and significant review, our team can pursue clarity and robustness. We sincerely appreciate your effort. We believe that the manuscript has been improved as per your recommendations. In accordance with all reviewers’ comments, statistical analysis was conducted again with robust manners.

**Comment 1**

The introduction predominantly relies on speculative links between biomechanics, psychological factors, and central sensitization. However, the study does not assess psychological or sensitization factors. I recommend a major revision of this section to focus more closely on the study's specific outcomes.

**Response:**

Thank you for your comment. Paragraphs regarding pectoral minor muscle and the relationship between lymph node resection and muscle tightness have been added to the Introduction.

(Page 4, line 60 – Page 5, line 70)

“However, the role of pectoral muscle dysfunction in neuropathic pain among BCS remains underexplored. Specifically, pectoralis minor muscle tightness may contribute to HLNP development. Pectoralis minor muscle tightness alters scapular biomechanics [17, 18] and reduces upper limb range of motion (ROM) [19, 20]. Consequently, repetitive movements with abnormal kinetic patterns can result in musculoskeletal conditions such as rotator cuff tears [21]. Furthermore, scapular depression caused by pectoralis minor muscle tightness can increase neural sensitivity [22] and directly compress or entrap nerves and blood vessels in the axillary region [23]. From a pain perspective, previous studies reported that postoperative pain may induce muscle hypertonicity [24]. Thus, we hypothesize that pectoralis minor tightness could serve as an indicator of HLNP, as the tightness may either cause postoperative pain or result from it.”

It has been updated to the limitation section that psychological and sensitization factors were not examined.

(Page 31, line 485 – 489)

“Fourth, the study did not include data on the contralateral side, preoperative physical assessments, and psychological evaluations of anxiety and depression, which are critical for comprehensive HLNP analysis. Finally, there was a limitation regarding measurements. We did not assess sensitization, which reduces the strength of our interpretation.”

**Comment 2**

Abbreviations: Please reduce the number of abbreviations, as the excessive use hinders readability.

**Response:** Thank you for your comment. Unnecessary abbreviations have been replaced with their full terminology—for example, neuropathic pain (NP), sentinel lymph node biopsy (SLNB), and axillary lymph node dissection (ALND).

**Comment 3**

Line 82: Specify whether you are referring to the pectoralis minor or major muscle.

**Response:**

Thank you for your comment. The muscle is the pectoralis minor muscles, and the relevant sentence is revised.

(Page 4, line 62 – 63)

“Pectoralis minor muscle tightness alters scapular biomechanics [17, 18] and reduces upper limb range of motion (ROM) [19, 20].”

**Comment 4**

Line 85: The hypothesis regarding lymph nodes is not supported by the introduction.

**Response:**

Thank you for your comment. The relevant paragraph has been incorporated into the Introduction section.(Page 5, line 71 – 79)

“Lymph node resection, a common procedure in breast cancer surgery, may influence postoperative musculoskeletal changes. Yang et al. [21] suggested that postoperative pain or radiation therapy could lead to pectoral muscle hypertonicity or fibrosis, no study has directly examined whether lymph node resection contributes to pectoralis minor muscle shortening.

Previous research has reported a marginally statistically significant negative correlation between shoulder external rotation range and the number of lymph nodes resected [25], suggesting that lymph node removal may impact musculoskeletal function. Given the anatomical proximity of lymphatic tissue to the pectoralis minor muscle [26], extensive lymph node resection may influence muscle length and consequently reduce upper limb ROM.”

**Comment 5**

Lines 117–118: Provide more details about the primary outcomes, including their psychometric properties between days.

**Response:**

Thank you for your comment. The paragraphs regarding PMI has been revised with detailed information.

(Page 9, line 160 – 175)

“The pectoralis minor length index (PMI) is a key independent variable in this study, quantifying pectoralis minor muscle tightness through standardized length measurements. While various methods and positions have been used to examine pectoralis minor muscle length in BCS [20, 31], this study measured the distance between the fourth intercostal space near the sternum and the coracoid process was measured in the supine position [20, 32, 33]. This position was selected to minimize the effect of gravity on posture [33] and was measured using a Palpation Meter (Performance Attainment Associates, St. Paul, MN, USA; Fig 2). Intra-rater reliability of the measurement in supine position was between 0.87 and 0.93 in patients with and without shoulder impingement symptoms [32]. The intra-tester reliability of the Palpation Meter was reported as ICC = 0.971, and it demonstrated good validity when compared with motion capture systems (r = 0.87) [31]. The measured length was standardized by participant height to calculate PMI using the formula: PMI = [(pectoralis minor muscle length/height) × 100] [34, 35]. Based on established criteria, a PMI value of 10 was considered indicative of normal resting muscle length [33]. To ensure measurement precision, three repeated assessments were conducted for each participant, with the average value used for subsequent analyses.”

**Comment 6**

Lines 119–120: In fact, the measurement used for PMI targets to estimate the distance between PM muscle origin and insertion and not scapular position. Please clarify.

**Response:**

Thank you for your comment. The statement regarding scapular position has been deleted.

(Page 9, line 160 – 161)

“The pectoralis minor length index (PMI) is a key independent variable in this study, quantifying pectoralis minor muscle tightness through standardized length measurements.”

**Comment 7**

Line 122: Why was this measured in the supine position, while the study mentions it was done standing?

**Response:**

Thank you for your comment. The reason for the measurement position has been added.

(Page 10, line 165 – 168)

“This position was selected to minimize the effect of gravity on posture [33] and was measured using a Palpation Meter (Performance Attainment Associates, St. Paul, MN, USA; Fig 2). Intra-rater reliability of the measurement in supine position was between 0.87 and 0.93 in patients with and without shoulder impingement symptoms [32].”

**Comment 8**

Line 123: Update reference 34 with the appropriate citation: Borstad JD, Ludewig PM. The effect of long versus short pectoralis minor resting length on scapular kinematics in healthy individuals. J Orthop Sports Phys Ther. 2005;35(4):227-38.

**Response:**

Thank you for your comment. The reference has been updated next to the PMI calculation, while the previous reference from Komati and colleagues remains, as they reported a PMI of 10 as the normal length.

(Page 9, line 170 – 172; References #34 and #35)

“The measured length was standardized by participant height to calculate PMI using the formula: PMI = [(pectoralis minor muscle length/height) × 100] [34, 35].”

34. Borstad JD, Ludewig PM. The effect of long versus short pectoralis minor resting length on scapular kinematics in healthy individuals. J Orthop Sports Phys Ther. 2005;35: 227-238. doi: 10.2519/jospt.2005.35.4.227, PubMed PMID: 15901124.

35. Borstad JD. Resting position variables at the shoulder: evidence to support a posture-impairment association. Phys Ther. 2006;86: 549-557. doi: 10.1093/ptj/86.4.549, PubMed PMID: 16579671.

**Comment 9**

Line 95: Was there no age limit for participants? If so, why?

**Response:**

Thank you for your comment. There was no age limit because we did not target a specific age group. Despite no age limitation, the age range was relatively young as immediate breast reconstruction is preferred to younger patients.

(Page 6, line 96 – 97)

“Given that IBR is typically recommended for younger patients, no upper age limit was established.”

**Comment 10**

Line 96: Clarify the recruitment setting.

**Response:**

Thank you for your comment. The recruitment setting has been updated. (Page 6, line 97 – 101)

“These patients were selected because their standard postoperative care included a follow-up visit at the rehabilitation medicine outpatient clinic approximately 1 month after surgery. Eligible patients were informed about the study during clinic visits and provided sufficient information to decide on participation.”

**Comment 11**

Lines 114–115: Add more details about the intervention and ensure that the discussion section uses the treatment delivered in this study rather than referencing previous studies with different techniques (see lines 330–333).

**Response:**

Thank you for your comment. Without referencing previous studies, details about the intervention have been updated. (Page 7, line 117 – 126)

“Participants were scheduled for physical therapy sessions. A.K. provided education and printed materials on pectoralis minor muscle stretching and scapular retraction exercises to all participants during the first educational session (S1 Appendix). Among the participants, 36 patients attended physical therapy for a median of three extra sessions (interquartile range [IQR]: 2–4 sessions), per the prescribed treatment plan. During these additional sessions, A.K. guided patients through shoulder elevation ROM exercises and administered deep frictional massage around the pectoralis minor muscles. Furthermore, 13 participants were prescribed self-care education sessions focused on edematous management, with a median of three sessions (IQR: 1–4 sessions), all conducted by a specialized physical therapist. Patients did not receive physical therapy such as thermoelectrical modality, to reduce pain.”

The discussion section was also updated. (Page 28, line 416 – 419)

“Despite the statistically insignificant differences, prevalence of HLNP decreased by 14%. We believe that our rehabilitation partially contributed to the reduction of HLNP prevalence during follow-up. A prior study initiated free ROM exercises 15 days postoperatively [50], whereas participants in this study began stretching around 30 days postoperatively..”

**Comment 12**

Lines 129–130: As primary outcomes, enough details should be provided in the manuscript itself rather than directing readers to previous studies.

**Response:**

Thank you for your comment. Paragraphs for outcomes are revised as in order of importance in this study.

(Page 8, line 130 – Page11, line 205)

“**Outcome measures**

This study employed both physical examinations and questionnaire-based measurements. The painDETECT questionnaire (PDQ) was conducted to evaluate HLNP, which was a dependent variable of the study, while independent variables included pectoral muscle length index (PMI), ROM, quality of life, functional symptoms, and upper limb disability.

**Dependent variable**

The presence and intensity of HLNP in the axillary and breast surgical regions were assessed using a validated painDETECT questionnaire (PDQ) developed based on neuropathic pain characteristics [28]. The PDQ is a validated tool for detecting neuropathic pain [7] and is widely used to identify neuropathic pain predictors in BCS [6, 11]. It consists of four parts: the first assesses pain intensity, while the remaining three parts evaluate pain location and characteristics. The total score is derived from the sum of the last three parts [28]. The PDQ demonstrates excellent discriminative validity, with established cutoff values: a score of 0–12 points indicates neuropathic pain is unlikely (< 15%), a score of 13–18 suggests neuropathic pain cannot be ruled out, and a score ≥19 indicates a high probability of neuropathic pain (> 90%) [28].

The original English version of the painDETECT questionnaire demonstrates a sensitivity and specificity of 85% and 80%, respectively, when differentiating neuropathic from nociceptive pain in patients with chronic low back pain. The Korean PDQ version used in our study has a sensitivity of 95.4% and a specificity of 73.8%, 13 points or lower to indicate nociceptive pain, with ≥19 points having a sensitivity and specificity of 95.4% and 73.8%, respectively, for neuropathic pain [29].

Considering that chronic pain is defined as pain lasting ≥ 3 months [30], HLNP was identified 4 months postoperatively. For primary analyses, HLNP was defined as a PDQ score ≥13, effectively distinguishing neuropathic pain from nociceptive pain [28]. Furthermore, HLNP was defined as a PDQ score ≥ 19 employed to differentiate neuropathic pain from both nociceptive and ambiguous pain.

Active ROM was evaluated for shoulder flexion, abduction, and internal and external rotation using a validated mobile application [36]. A mobile phone was attached to the operated arm using an armband. Participants were asked to raise the arm in sagittal and frontal plane in a sitting position to examine active ROM for flexion and abduction, respectively. Subsequently, participants were asked to lie on the examination bed, with their arms abducted to 45˚ and the elbow flexed to 90°, while the mobile phone remained attached to lower arm during external and internal arm rotation. The reliability of ROM assessments was > 0.9 [27]. Three repetitions were conducted, and average data was used in the study. Pectoral tightness was defined as an average forward flexion and abduction < 170°, combined with external rotation > 80° [21], and tightness of the pectoralis major muscle.

Pain intensity was measured using the 11-point numeric rating scale (0 to 10) embedded within the PDQ. Participants reported their current pain intensity, strongest pain experienced in the last 4 weeks, and the average pain during the same period. Quality of life, functions, and symptoms were assessed using the Korean versions of the European Organization for Research and Treatment of Cancer Quality of Life Questionnaire Core 30 and the breast cancer-specific questionnaires [37, 38], following their respective manuals [39, 40]. Symptom scales included fatigue, insomnia, pain, arm and breast symptoms, body image, and systemic therapy side effects (ST). Each symptom was evaluated through multiple items [39, 40]. For instance, the ST scale includes seven items assessing symptoms such as dry mouth, altered taste, eye pain, hair loss, general illness, hot flashes, and headaches. Both questionnaires demonstrated good validity and reliability in BCS [41]. Upper limb disability was measured using the Quick Disabilities of the Arm, Shoulder, and Hand (Quick-DASH) questionnaire [42], which has demonstrated acceptable validity and high level of reliability in BCS [43], and responsiveness in upper limb patients [44]. Studies reported a Cronbach’s alpha of 0.93 and test-retest reliability of 0.78, and this tool was validated to use in BCS. The Korean version of the questionnaire was used [45], and permission to use all questionnaires was obtained from their respective official sources.”

**Comment 13**

Line 142: Relying on patient memory to rate the strongest pain over a four-week interval introduces bias, as patient recall is not a reliable tool, especially over time. Please address this in the limitations section. Table 2 also suggests potential bias in the HLNP group (score = 5.5, only for this measurement).

**Response:**

Thank you for your comment. This is added to the limitations section.

(Page 31, line 491 – 492)

“Furthermore, some questions relied on the patient's memory, potentially introducing recall bias.”

**Comment 14**

Line 153: The abbreviation "Quick-DASH" is more commonly used than "Q-DASH."

**Response:**

Thank you for your comments. Q-DASH has been revised to Quick-DASH throughout the manuscript, including all four instances—table captions included.

**Comment 15**

Line 154: The responsiveness demonstrated in study 44 has not been validated in a cancer population; please address this.

**Response:**

Thank you for your comment. The statement regarding reliability, validity, and responsiveness has been updated. (Page 11, line 199 – 203)

“Upper limb disability was measured using the Quick Disabilities of the Arm, Shoulder, and Hand (Quick-DASH) questionnaire [42], which has demonstrated acceptable validity and high level of reliability in BCS [43], and responsiveness in upper limb patients [44]. Studies reported a Cronbach’s alpha of 0.93 and test-retest reliability of 0.78, and this tool was validated to use in BCS.”

**Comment 16**

Statistical analysis: The manuscript lacks details regarding sample size estimation.

Line 161: Clarify which groups are being referenced. Based on the results, I assume the groups were defined by neuropathic pain, but this is unclear in the methods section.

**Response:**

Thank you for your comment. Sample size estimation was included in the Materials and Methods section. (Page 13, line 235 – 239)

“Sample size calculations and post-hoc power analysis were conducted using G*power software (latest version 3.1.9.7; Heinrich-Heine-Universität Düsseldorf, Düsseldorf, Germany). The power analysis determined that at least 42 participants would be required (OR: 3; α = 0.05; power = 0.8; and probability of Y: 0.5). Therefore, the final sample size was sufficient to run this analysis.”

We have clearly defined the target group in the previous paragraphs and explicitly stated the basis for comparisons. (Please refer to Comment 12)

**Comment 17**

Line 185: Why is this data important for the study population? Would patients with diabetes have been excluded?

**Response:**

Thank you for your comment. We admitted including diabetes mellitus as exclusion criteria. The sentence in the Results section was removed. Relevant description has been inserted in the Materials and Methods section. (Page 6, Line 105)

“(6) diabetes mellitus (to exclude confounding by diabetic neuropathy)”

**Comment 18**

Table 2: Clarify why the Non-HLNP group presented more patients with pectoral tightness but less pectoralis minor shortening, as well as why this group demonstrated 16 degrees more abduction ROM compared to the HLNP group. This requires further explanation in the discussion.

**Response:**

Thank you for your valuable comment. We sincerely apologize for the classification error. The current version of the data and tables has been corrected, and the relevant regression analysis results have also been revised accordingly.

**Comment 19**

Lines 212–219: The odds ratios (ORs) reported are close to 1, with confidence intervals that cross 1 or are very wide (e.g., CI = 0.046–0.74). While these results are statistically significant, are they clinically meaningful? Please integrate this discussion into the main findings and conclusions.

**Response:**

Thank you for your comment. Statistically significant variables are re-evaluated with robust manners. Relevant description has been revised, and the discussion has been updated.

(Page 27, Line 394 – 403) “Despite the wide confidence interval of PMI in both HLNP models, we consider PMI a clinically and statistically relevant indicator of HLNP prevalence. The results indicate that higher preceding pain intensity is associated with a lower PMI, while a higher PMI correlates with greater shoulder abduction. Pain intensity was omitted from the final analysis; however, these findings underscore the role of acute pain in PMI changes and HLNP development. Given the weak correlations between PMI and current pain intensity, we propose that BCS adopt protective slouched postures due to acute postoperative pain [21]. Considering the wide confidence interval of PMI, a lower PMI, as a reaction to pain, may serve as a preceding factor for HLNP presence. However, further validation with larger sample sizes is necessary to confirm these associations.”

(Page 18, Line 435 – 438) “ST was statistically significant; however, its modest odds ratio (1.056) and low statistical power suggest that its predictive strength is limited. Therefore, its association with insomnia, fatigue, and arm symptoms indicates that it may play an indirect role in HLNP development.”

**Comment 20**

Lines 218–219: Why did you decide to present this result? The study's primary focus was on a PDQ score ≥ 13. Please clarify.

**Response:**

Thank you for your comments. Although HLNP was defined as a PDQ score ≥ 13, an alternative cutoff value was identified for sensitivity analysis. Relevant sentences have been added to the Materials and Methods section. As a result of the sensitivity analysis, the findings consistently indicate that a lower PMI is associated with a higher prevalence of HLNP. However, PDQ≥19 criterion can be omitted if it is deemed redundant or noisy.

(Page 12, Line 222 – 228) “Sensitivity analysis was performed under two conditions: (1) incorporating time since surgery to enrollment as a covariate for multivariate analysis, accounting for its impact on physical recovery (ranging from 26 to 57 days due to the study’s clinic schedule), and (2) applying an alternative HLNP definition (PDQ score ≥19 [11]). The PDQ ≥19 threshold reliably distinguishes definitive neuropathic pain from nociceptive/ambiguous pain and has been validated in previous BCS pain studies [6, 11]. Both the two-step logistic regression and covariate-controlled multivariate regression were repeated for this alternative definition.”

**Comment 21**

Table 3: Why present both cutoff values? The primary focus was on a PDQ score ≥ 13. **Response:**

Thank you for your comments. As you mentioned, the primary and secondary focus were displayed together to avoid redundancy. Creating a separate table for the secondary focus was deemed unnecessary; however, the PDQ ≥ 19 criterion can be omitted if it is considered redundant. (Please refer to the Comment 20)

**Comment 22**

Table 3: Highlight the wide confidence intervals for PMI in the discussion.

**Response:**

Thank you for your comment. Relevant sentences have been added.

(Page 18, line 299 – 301) “While current pain intensity showed consistent estimates (OR: 1.4; CI: 0.9 – 2.0), PMI’s wide confidence interval warrants careful interpretation.”

(Page 27, line 394 – 395) “Despite the wide confidence interval of PMI in both HLNP models, we consider PMI a clinically and statistically relevant indicator of HLNP prevalence.”

**Comment 23**

Table 3: The systemic therapy side effect results were modest. Are these findings clinically important?

Lines 235–236: Again, discuss the discrete results and large confidence intervals (Table 4) in the discussion.

**Response:**

Thank you for your comment. As a predictor or preceding factor, it can be considered clinically important. However, the modest OR with statistical significance must be highlighted.

(Page 17, line 280 – 283) “The most significant predictor was ST, with an OR of 1.058 (95% CI: 1.017–1.100, *p* = 0.005). We consider the role of this predictor or preceding factor for HLNP prevalence as clinically significant; however, further interpretation requires caution on the modest OR effect size.”

(Page 29, line 434 – 445) “ST was statistically significant; however, its modest odds ratio (1.056) and low statistical power suggest that its predictive strength is limited. Therefore, its association with insomnia, fatigue, and arm symptoms indicates that it may play an indirect role in HLNP development. While no direct correlation was found between ST score and pain severity, ST was statistically significantly associated with symptoms such as insomnia, fatigue, arm symptoms, pain, and body image subscale scores from the questionnaires. Given the significant interconnection between insomnia, fatigue, and pain [54], and the negative impact of pain—including arm symptoms—on body image [55], a higher ST score may precede pain chronification. Since chemotherapy is the primary factor influencing ST scores, particular attention should be given to the side effects experienced by BCS who have undergone chemotherapy.”

**Comment 24**

Line 237: Add this analysis to the statistical analysis section.

**Response:**

Thank you for your comment. Relevant sentences have been added and revised to the Methods section.

(Page 12, line 227 – 228) “Both the two-step logistic regression and covariate-controlled multivariate regression were repeated for this alternative definition.”

**Comment 25**

Lines 240–241: The small sample size is a study limitation. The reader needs to have clarity that the results were not large and that the sample was small for the number of variables included in the regression analysis.

**Response:**

Thank you for your comment. The previous section has been deleted to balance the statistical significance and small sample size. The results of multivariate regression analysis have been updated with post-hoc analysis. The limitation identified the influence of small sample size of the study.

(Page 22, line 318 – 322) “The post-hoc power analysis indicated power as 0.05 (OR: 1.056, α = 0.05, n = 57, probability of Y = 0.38) for ST. For PMI, the power reached 0.99 (OR: 4.9, calculated as 1/0.204). Similar to the univariate regression results, both factors require careful interpretation due to their modest ORs and wide confidence intervals.”

(Page 22, line 328 – 329) “Post-hoc analysis reported a power of 0.99 with an OR of 8.62 (1/0.116), α = 0.05, sample size = 57, and probability of Y = 0.14.”

(Page 32, line 478 – 480) “The small sample size contributed to the wide confidence interval for the OR of PMI and the significant but very low OR of ST, both of which reduced the statistical power of our analyses.”

**Comment 26**

Lines 250–257: Congratulations on adding this analysis—it is practical for clinicians and future research. Well done!

**Response:**

We sincerely appreciate your kind words and encouragement. Your positive feedback is truly motivating, and we are grateful for your support.

**Comment 27**

The discussion focuses heavily on sensitivity and sensitization. Why were these not assessed in the study? This should be acknowledged as a limitation.

**Response:**

Thank you for your comment. Paragraphs regarding sensitivity and sensitization have been revised.

(Page 27, line 404 – 414) “Biomechanically, a reduced PMI indicates scapular depression and protraction, restricting pectoralis major muscle mobility during arm elevation [17]. These biomechanical alterations may induce abnormal movement sensations and soft tissue injury around the shoulder [21]. Chronic pain development in this context likely involves central sensitization, while scapular malalignment exacerbates peripheral sensitization through pectoral nerve compression [23]. Furthermore, scapular malalignment has been linked to increased local pain pressure sensitivity [22], a hallmark of peripheral sensitization. These findings corroborate prior studies linking chest tightness and upper limb discomfort [20], supporting scapular dysfunction as a risk factor for HLNP. Since we did not measure sensitization properties, this hypothetical interpretation calls for further studies that use specific measurements such as quantitative sensory testing.”

Not conducting those properties has been included in the Limitations.

(Page 31, line 490 – 491) “Finally, there was a limitation regarding measurements. We did not assess sensitization, which reduces the strength of our interpretation.

**Comment 28**

Lines 287–289: Is ST considered a physical parameter? Based on Table 2, side effects seem to have influenced HLNP. How could this be evaluated earlier?

**Response:**

Thank you for your comments. ST is a subjective parameter. It can be measured using questionnaires, EORTC BR-23, which is most common questionnaires in medical practice for cancer patients.

(Page 27, line 386 – 387) “PMI can be readily measured using specific instruments or calipers, while ST can be assessed using global questionnaires.”

The term ST is defined in the Methods section. The relevant sentence has been added to the end of paragraphs.

(Page 11, line 197 – 198) “For instance, the ST scale includes seven items assessing symptoms such as dry mouth, altered taste, eye pain, hair loss, general illness, hot flashes, and headaches.”

**Comment 29**

Lines 310–312: This sentence does not align with the paragraph's context and requires further development.

**Response:**

Thank you for your helpful comments. The sentence in question has been deleted, and the entire paragraph has been revised accordingly (please refer to the Comment 27).

**Comment 30**

Lines 313–314: Be cautious with your claims; the study did not assess the pectoral fascia.

**Response:**

Thank you for your comments. The paragraph has been withdrawn.

**Comment 31**

Lines 342–344: The transitions are abrupt, and this sentence is underdeveloped. Expand on this idea and connect it to your findings.

**Response:**

Thank you for your helpful comments. The sentence in question has been deleted, and the entire paragraph has been revised accordingly (please refer to the Comment 23).

**Comment 32**

Lines 350–355: Further discussion is needed. Instead of simply comparing your results with previous studies, emphasize the clinical impact and relevance of your findings.

**Response:**

Thank you for your comments. The paragraph has been revised to move beyond a simple comparison of our results with previous studies and instead emphasize the clinical impact based on our findings. (Page 29, line 446 – 464)

“Another novel observation was the interaction between the amount of resected lymph nodes and physical variables influenced by reconstruction type and adjuvant therapy. Our results partially support the hypothesis of a significant association between the number of lymph node resections and PMI. Although the difference was not statistically significant due to the imbalance in sample size (52 vs. 5), PMI was lower in BCS who underwent only axillary lymph node dissection compared with those who had a sentinel lymph node biopsy. Furthermore, the correlation test for the sentinel lymph node biopsy group supports this hypothesis. Given that the axillary lymph nodes are anatomically located near the pectoralis minor muscle, the extent of axillary surgery may contribute to pectoral minor muscle length changes, potentially affecting shoulder mobility and upper limb function. These findings highlight the clinical importance of postoperative musculoskeletal assessment in BCS undergoing axillary surgery.

Surgical differences appear to significantly influence this relationship. Implant-based reconstruction, requiring significant soft tissue excision, muscle detachment, and material anchoring, could intensify postoperative pain and prolong pectoral minor muscle recovery [56]. Conversely, transverse rectus abdominis flap reconstruction prevents abnormal muscle activation patterns as this approach does not require significant soft tissue excision and preserves the pectoral major muscle. Instead, it fills the empty spaces with donor-site adipose tissue, potentially reducing muscular tightness and postoperative discomfort [24].”

To support this discussion, we conducted additional analyses to explore the relationships between variables. The relevant details have been provided in the Results section. (Page 24, line 355 – 369).

“Spearman’s rank correlation assessed relationships between the number of resected lymph nodes and physical variables (Table 5). In BCS who underwent sentinel lymph node biopsy, an increased number of resected lymph nodes was associated with reduced external rotation and decreased PMI. The type of breast reconstruction and adjuvant therapy also influenced these associations. ST scores were statistically significantly higher in patients who underwent chemotherapy compared to those who did not (median: 23.81 vs. 14.29, p = 0.017). ST demonstrated statistically significantly correlations with insomnia (ρ = 0.602, p < 0.001), fatigue (ρ = 0.559, p < 0.001), arm symptoms (ρ = 0.430, p = 0.001), pain (ρ = 0.329, p = 0.012), and body image (ρ = -0.327, p = 0.013). Furthermore, PMI was positively correlated with shoulder abduction (ρ = 0.297, p = 0.013) and negatively correlated with arm symptoms (ρ = -0.353, p = 0.004) and average pain intensity over the preceding month (ρ = -0.279, p = 0.018). PMI was higher in BCS who underwent sentinel lymph node biopsy (N = 52) compared with those who underwent only axillary lymph node dissection (N = 5); however, the difference was not statistically significant (median: 9.98 vs. 9.80, p = 0.337). Correlation coefficients between these variables are provided in S2 Table.”

**Comment 33**

Lines 384–386: The results do not substantiate this speculation.

**Response:**

We sincerely appreciate your insightful comments. The overstated sentences have been carefully removed to improve the clarity and accuracy of the manuscript.

This is new conclusion (Page 31, line 493 – 504)

“This study identified the PMI and ST at 1 month postoperatively as significant predictors of HLNP at 4 months following mastectomy with lymph node resection. PMI < 9.82 and ST > 23.81 were associated with an elevated risk of HLNP, a condition significantly correlated with upper limb functional impairment. Both predictors were significantly correlated with acute pain and its chronification. These findings underscore the critical need for early postoperative evaluations to identify patients at heightened risk for NP and associated functional limitations. Multidisciplinary postoperative care plans incorporating comprehensive symptom assessments targeted physical interventions, and close monitoring within the first-month post-surgery are strongly recommended to mitigate HLNP risk. Future research should focus on larger cohorts and randomized controlled trials to validate these findings by comparing the effects of early initiation of physical therapy to increase PMI versus usual care.”

**Comment 34**

Line 389: Clarify how RCTs could validate these results.

**Response:** Thank you for your comments. The future study direction has been clarified, specifying an RCT design comparing early physical therapy to increase PMI versus early pain medication prescription.

(Page 32, line 501 – 504)

“Future research should focus on larger cohorts and randomized controlled trials to validate these findings by comparing the effects of early initiation of physical therapy to increase PMI versus usual care.”

**Comment 35**

Line 390: The type of study conducted does not support this claim.

**Response:**

Thank you for your comments. The sentence has been deleted.
