# Supplementary material for: Pectoralis minor length index at 1 month postoperative can predict homolateral neuropathic pain 4 months after mastectomy with lymph node resection
Source: PLoS One. 2025 Jun 12;20(6):e0326119. doi: 10.1371/journal.pone.0326119 (PMC12161585; doi:10.1371/journal.pone.0326119)
Supplement: Attachment — Submitted filename: 9._Response_to_Reviewers_250520.docx [file pone.0326119.s007.docx]

**Minor revision decision**

The major concerns of the reviewers have been addressed. However, there still remains some minor issues that need to be resolved in my perspective as an academic editor.

I believe that the rationale behind this work is innovative but I agree with reviewer 3 that third paragraph of the introduction is confusing. This is not only because it introduces pectoralis minor (PM) tightness as a “wellproven” hypothesis that explains HLNP but also because it is not consistent with the limitations raised by reviewer 3.

**Response:**
Thank you for your thoughtful and thorough review. Please find below our point-by-point responses addressing each of your comments.

In the meantime, I may send you one further peer review to ensure that overstated points in the paragraph 3 will be rewritten in a more skeptical tone, mainly regarding:

**Comment 1**

How PM changes post-breast cancer surgery might influence neuropathic pain development because (reference 22) does not mention that scapular depression is not a proven consequence of PM tightness.

**Response:**
Thank you for your valuable comments. Paragraph 3 has been revised to more clearly articulate the background rationale explaining how pectoralis minor (PM) changes following breast cancer surgery may influence HLNP development. Additionally, the sentence referencing Reference 22 has been revised to directly address your concern that scapular depression is not a proven consequence of PM tightness.

The updated version of Paragraph 3 is provided below (Page 3, new lines 60–75):

“However, the role of pectoral muscle dysfunction in the development of neuropathic pain among BCS remains underexplored. Specifically, pectoralis minor muscle tightness may contribute to HLNP development through its impact on surrounding tissues. Pectoralis minor muscle tightness alters scapular biomechanics [17, 18] and reduces upper limb range of motion (ROM) [19, 20]. Consequently, repetitive movements with abnormal kinetic patterns can result in musculoskeletal conditions such as rotator cuff tears [21]. Lee et al. [20] identified chest tightness, assessed via pectoralis minor muscle length, as a contributing factor to upper limb pain after breast cancer surgery. From a pain perspective, previous studies have reported that postoperative pain may induce muscle hypertonicity [22]. Altered muscle activation patterns caused by anterior chest wall tightness may reflect protective responses to pain arising from irritation of injured tissues. Furthermore, scapular depression has been linked to increased neural sensitivity [23] and possible compression or entrapment of nerves and blood vessels in the axillary region [24]. Taken together, these findings suggest that pectoralis minor tightness may be a consequence of both tissue damage and a contributing factor to neural irritation. Thus, we hypothesize that pectoralis minor tightness could serve as an indicator of HLNP, as the tightness may either cause postoperative pain or result from it.”

Additionally, the **limitations section** has been updated to reflect this issue (Page 32, new lines 498–501):

“Finally, there was a limitation regarding measurements. We did not assess sensitization, which reduces the strength of our interpretation. Consequently, we could not fully evaluate the underlying rationale for how pectoralis minor muscle tightness may contribute to HLNP development.”

**Comment 2**

There is no established cutoff value for PM tightness in clinical populations. Therefore, this a must written limitation that the authors must state to justify the use of values from young, asymptomatic individuals (reference 18). Also, this point needs to be stated again in the limitations section in details.

**Response:**

Thank you for your valuable comment. Relevant statements have been added to both the Results section and the Limitations section to address the lack of a validated cutoff value for pectoralis minor tightness in clinical populations.

Pages 10-11, new lines 177–179

“Based on established criteria, a PMI value of 10 was considered indicative of normal resting muscle length in young, asymptomatic individuals [33]. However, to date, there is no published literature reporting a normative PMI value in BCS.”

Page 32, new lines 493–496

“Due to the lack of existing literature, a normative PMI cutoff value of 10 in young, asymptomatic individuals was referenced [33]. However, this comparison should be re-evaluated once a standardized PMI cutoff for the operated side in BCS is established.”
